# Therapeutic Notes

**Published:** 1903-09

**Authors:** Nelson W. Wilson

**Affiliations:** Buffalo, N.Y.


### Therapeutic Notes.

CONDUCTED BY NELSON W. WILSON, M. D., Buffalo, N.Y.

ARSENIC IN TUBERCULOSIS.

*Le Journal de Medicine de Paris,* in an extensive article on tuberculosis, lays considerable stress upon the use of arsenic in the early stage of the disease and in the chloro-anemic forms common in young women. The best way to administer it is with the hypodermic needle from 5 to 10 centigrams to the cubic centimeter being given once a day. When given by mouth it is best to administer it in concentrated form:

| | | | |
|---|---|---|---|
| ℞ | Cacodylate of soda | 0.40 | gr. vii. |
| | Distilled water | 10.00 | ʒii. ss. |

If a pill is preferred it would be best given thus:

| | | | |
|---|---|---|---|
| ℞ | Cacodylate of soda | 0.025 | gr. 1-3 |
| | Ext. gentian | 10.0 | ʒii. ss. |

For one pill.

In some cases the stomach will be so sensitive that these prescriptions cannot be borne. In such cases administer by rectum:

| | | | |
|---|---|---|---|
| ℞ | Cacodylate of soda | 0.40 | gr. vii. |
| | Distilled water | 180.00 | ℥vi. |

Directions: Inject 5 c.c., twice daily.

Generally speaking the treatment should be intermittent, hypodermics daily for 8 days and suspended for 8 days; injections 2 or 3 times weekly.

---

### FOR ACUTE ALCOHOLISM.

VERY often in the course of an everyday practice one receives a call from a gentleman—or otherwise—who is suffering from acute alcoholism. He wants to be "fixed up." J. M. Anders gives such cases this:

| | | | |
|---|---|---|---|
| ℞ | Bromide of soda | 30.0 | ℥i. |
| | Tr. capsicum | 4.0 | ʒi. |
| | Tr. digitalis | 2.0 | ʒss. |
| | Simple elixir to make | 60.0 | ℥ii. |

Mix—Dose: Teaspoonful every 2 or 3 hours in water.

---

### IN BOTH KINDS OF HEMORRHOIDS.

FROM Paris comes this formula for external hemorrhoids:

| | | | |
|---|---|---|---|
| ℞ | Chrysarobin | 0.8 | gr. xii. |
| | Iodoform | 0.3 | gr. v. |
| | Ext. belladonna | 0.6 | gr. x. |
| | Vaseline | 20.0 | ʒ v. |

For the more retiring type of hemorrhoids, the internal, this is best:

| | | | |
|---|---|---|---|
| ℞ | Chrysarobin | 0.08 | gr. i 1-3 |
| | Iodoform | 0.08 | gr. i 1-3 |
| | Ext. belladonna | 0.01 | gr. 1-6 |
| | Coca butter | 2.00 | ʒ ss. |

After using for a few days pain and hemorrhage disappear and in from 3 to 5 months the hemorrhoids have entirely retracted. Before each application of either of the above there should be thorough disinfection with a solution of carbolic acid.

